# In-vitro investigation of bone temperature changes in osteotomies performed with different brands of implant burs

**DOI:** 10.1186/s40729-025-00588-9

**Published:** 2025-02-11

**Authors:** Ömer Faruk Şarkbay, Ahmet Mihmanli, Hakan Cora

**Affiliations:** 1 Istanbul Gelisim University-Vocational School of Health, Istanbul, Türkiye; 2 Istanbul Gelisim University-Faculty of Dentistry, Istanbul, Türkiye; 3 Maltepe University-Faculty of Management, Istanbul, Türkiye

**Keywords:** Implant, Dentistry, Temperature, Bone, Osteotomy

## Abstract

The aim of this study was to investigate in-vitro the temperature changes occurring in the bone during drilling with implant drills manufactured by different companies. Bone blocks obtained from fresh bovine ribs were used in the study. Bone blocks were drilled with drills manufactured by Ankylos, Astra Tech, Nobel Biocare, Bredent and Straumann implant brands at an ambient temperature of 30 ± 2° C under a constant pressure of 2 kg. Two K-type thermocouple sensors were placed on the bone blocks at 5th and 10th mm depths and the temperature changes were measured at a distance of 1 mm from the implant drill. In the study, working models were created under different conditions for implant socket preparation. In group 1, the first time drills were used at 150 rpm without irrigation, in group 2, the first time drills were used at 1200 rpm with 40 ml/min irrigation, in group 3, the 30th time drills were used at 150 rpm without irrigation, and in group 4, the 30th time drills were used at 1200 rpm with 40 ml/min irrigation. All osteotomy procedures were performed with 3.5 ± 0.3 mm diameter burs for a period of 8 s and the temperature values obtained at equal time intervals were recorded. Repeated Measures and Kruskall Wallis-H tests were used for statistical analysis of the data. No significant difference was observed between the implant drills and the temperature changes in the bone during drilling (p < 0.05). None of the groups reached critical temperature values (47° C+) throughout the study. At the 5th and 10th mm depths, the temperature changes in the sensors used were close to each other. It was also calculated that although the average temperatures were close to each other in the non-irrigated and irrigated systems, the difference values obtained by subtracting the initial temperature were significantly higher in the non-irrigated systems. The results showed that implant drills did not cause significant temperature increases in bone blocks depending on the difference in manufacturers (Ankylos, Astra Tech, Nobel Biocare, Bredent, Straumann) and the number of uses. It was also concluded that irrigated and non-irrigated systems are safe as long as they are used under the recommended conditions.

## Introduction

The surgical stage plays an important role in the success of dental implant applications. For this reason, researchers have focused on safe surgical procedures performed on the jaw bones during dental implant placement [1–3]. At the same time, it has been emphasized that the surgical techniques used are important not only in ensuring successful osteointegration but also in the prognosis of the implants placed [4, 5]. It is known that protein denaturation occurs at high temperatures in living tissues [6, 7]. In the literature, the effect of temperature changes in the bone during osteotomy with drilling on osteointegration and thus on implant success has been a subject of debate [8, 9]. At this stage, it is thought that many factors such as the material from which the implant drills are made, bone morphology in which the implant socket is prepared, implant groove structure, working speed, amount and temperature of irrigation solution may be effective [10, 11].

In this in-vitro study, it was aimed to compare the temperature changes in the bone caused by irrigated and non-irrigated systems at different speeds (150 rpm and 1200 rpm) during drilling with implant drills produced by five different implant brands (Ankylos, Astra Tech, Nobel Biocare, Bredent, Straumann).

## Materials and methods

### Findings

This study is derived from a master's thesis. In the study, the temperature changes in the bone caused by the burs of 5 different implant brands were evaluated and measured under different working conditions and at different usage numbers.

### Measured average temperature values

#### First group

In this group, waterless operation was performed at 150 rpm and the milling cutters were used for the first time. The average highest and lowest temperature values obtained as a result of the experiment show the average temperature values observed in the first working group. T_max_:The highest temperature, T_min_:The lowest temperature, T_ort_:The average temperature during the working time, SD:Standard deviation, _Tmax-min_:The difference between the highest and lowest temperatures.

#### Second group

In this group, 40 ml/min irrigation at 1200 rpm was used and milling cutters were used for the first time. The average highest and lowest temperature values obtained as a result of the experiment are shown in the average temperature values observed in the second study group. T_max_:Highest temperature, Tmin:Lowest temperature, T_ort_:Average temperature during the study period, SD:Standard deviation, _Tmax-min_:Difference between the highest and lowest temperatures.

#### Group three

In this group, irrigation was not used at 150 rpm and milling cutters that had been used 30 times before were used. The average highest and lowest temperature values obtained as a result of the experiment are shown in the average temperature values observed in the third working group. T_max_:Highest temperature, T_min_:Lowest temperature, T_ort_:Average temperature during the working period, SD:Standard deviation, _Tmax-min_:Difference between the highest and lowest temperatures.

#### Group four

In this group, the milling cutters that had been used 30 times before were used under 40ml/min irrigation at 1200 rpm. The average highest and lowest temperature values obtained as a result of the experiment are shown in the average temperature values observed in the fourth working group. Tmax: Highest temperature, Tmin: Lowest temperature, Tort: Average temperature during the working period, SD: Standard deviation, Tmax-min: Difference between the highest and lowest temperatures.

**Fig. 1:**
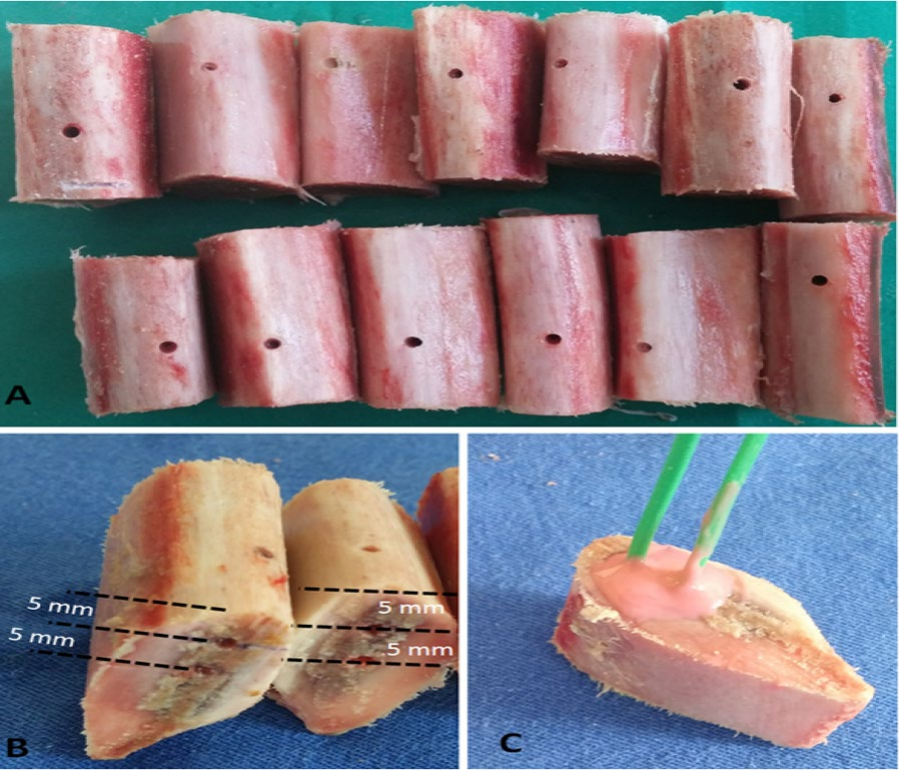
View of guide slots (**a**), opening of standard sensor slots from heights of 5 and 10 mm (**b**), placing and fixing thermocouple sensors (**c**)

After the preparation of the bone blocks, the areas where osteotomy would be performed were determined on the bone. Guide implant holes with standard diameters of 2 mm were created in these determined areas using the marking bur and pilot bur used as standard in implant site preparation (Fig. 1a). Then, the holes where the thermocouple sensors that would measure the temperature changes in the bone-bur contact areas at 5 and 10 mm would enter were prepared (Fig. 1b). The thermocouple sensors placed in the prepared holes were fixed with a silicone-based impression material (Fig. 1c).

**Fig. 2:**
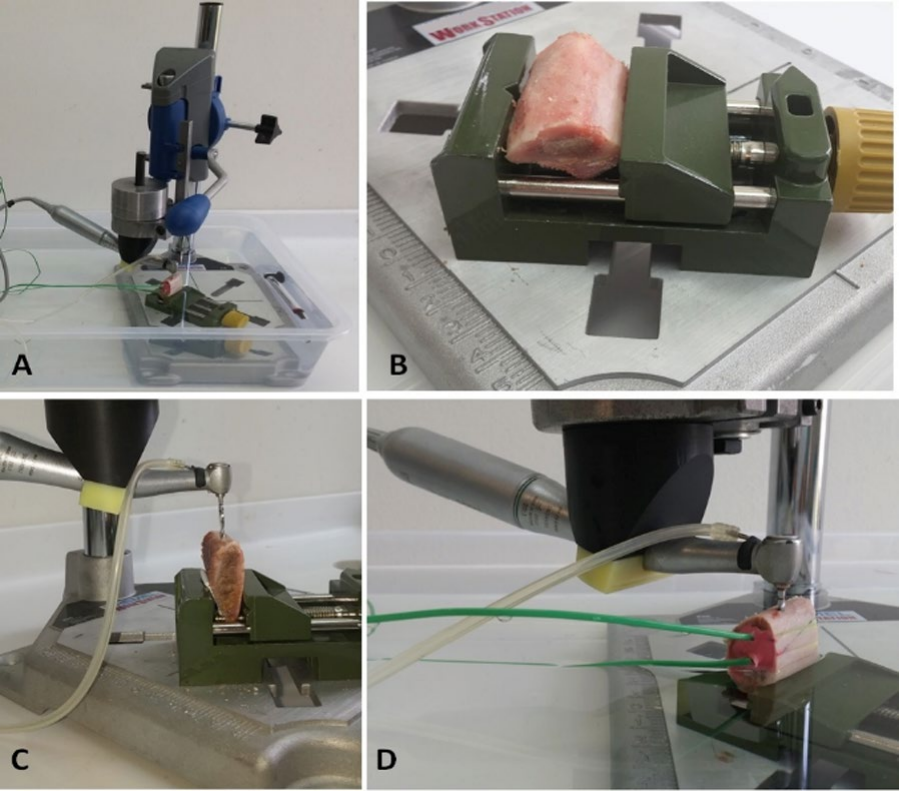
General view of the contra-angle holder system (**a**), fixation of bone blocks (**b**), opening of guide slots (**c**) and operation of the experimental setup (**d**) are shown

**Fig. 3:**
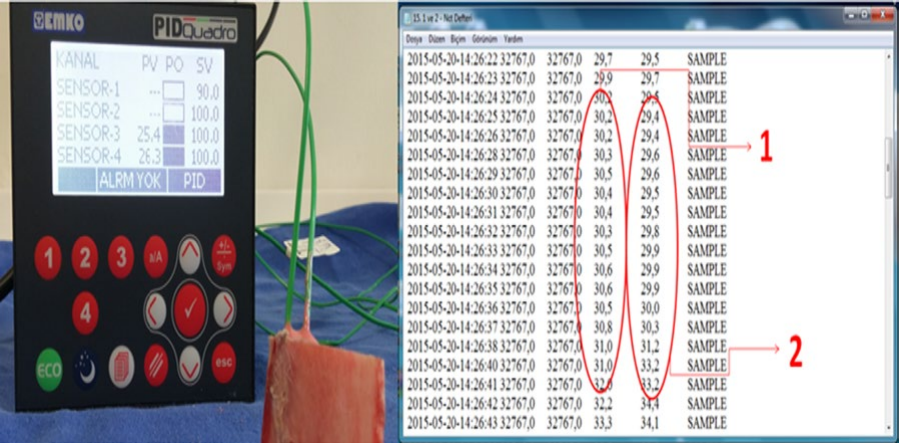
Data transferred to the computer via thermocouple (EPLC9600-PID QUADRO) connection and flash memory; Data number 1 shows the temperature values at a depth of 5 mm, and data number 2 shows the temperature values at a depth of 10 mm

A specially manufactured system was used in the preparation of implant sockets (Fig. 2a). After the blocks were placed in the bone holder compartment in the system, they were tightened and fixed (Fig. 2b). The system was operated under constant pressure (with a 2 kg weight). Figure 2c shows the preparation of 2.0 mm sockets using implant guide burs. During the experimental phase, the container containing the device was filled with 30 ± 2 C0 water in order to keep the temperature constant. The temperature of the system was controlled with the water heater and thermometer placed. The temperature values in the bone were recorded with thermocouple sensors placed in the bone block (Fig. 3d).

### Difference between the highest and lowest temperature values

The values observed at the sensors located at the 5th and 10th mm (S1 and S2, respectively) placed in the bone blocks and obtained by subtracting the lowest temperature value from the highest temperature value are shown in Table 1.

**Table 1:** Differences between different brands of implant burs and the highest and lowest temperatures recorded in the study

	Ankylos			Astra Tech		Nobel Biocare		Bredent		Straumann	
Group No	S_1_	S_2_		S_1_	S_2_	S_1_	S_2_	S_1_	S_2_	S_1_	S_2_
1	7,9	4,7		12,3	2,3	7,8	5,7	16,0	11,2	5,9	14,8
2	8,9	11,5		10,9	12,1	6,0	9,4	6,6	6,3	9,6	3,2
3	6,7	6,2		10,8	6,0	10,7	3,5	5,9	8,8	12,6	14,1
4	6,6	6,3		1,8	3,8	4,7	8,1	4,3	2,3	7,5	8,2

### Relationship between measured temperature values and working groups

There was no significant relationship between the measured temperature values and the study groups. The results of the statistical analysis are given in Table 2.

**Table 2:** Relationship between study groups in different conditions + Pillai's Trace test and Bonferroni analysis (Group 1: First use, without irrigation, 150 rpm; Group 2: First use, 40 mL/min irrigation, 1200 rpm; Group 3: 30th use, without irrigation, 150 rpm; Group 4: 40 mL/min irrigation, 1200 rpm

Working groups	^+^p
Group 1	
Group 2	158
Group 3	1000
Group 4	267
Group 2	
Group 1	158
Group 3	074
Group 4	1000
Group 3	
Group 1	1000
Group 2	074
Group 4	131
Group 4	
Group 1	267
Group 2	1000
Group 3	131

No significant correlation was observed between the mean temperature values observed in the study groups and implant brands. The results of the statistical analysis are given in. During the study, no significant difference was observed between the mean temperature values of cases irrigated at 1200 rpm (Group 2 and Group 4) and cases irrigated at 150 rpm (Group 1 and Group 3) (Independent T-test; p=0.682)

### Calculation of temperature differences

The temperature changes in all brands in the study were obtained by subtracting the lowest temperature value measured during the study from all temperature values (Tables 3 and 4).

**Table 3:** Relationship between temperature changes and implant brands^+^ Pillai's Trace and Bonferroni tests

BRAND	^+^p	BRAND	^+^p	BRAND	^+^p
Ankylos	Astra	708	Astra	Ankylos	708
	Nobel	1000		Nobel	936
	Bredent	752		Bredent	1000
	Straumann	1000		Straumann	995
Nobel	Ankylos	1000	Bredent	Ankylos	752
	Astra	936		Astra	1000
	Bredent	940		Nobel	940
	Straumann	1000		Straumann	991
Straumann	Ankylos	1000			
	Astra	995	Repeated measures p < 0.05		
	Nobel	1000			
	Bredent	991			

When compared according to the number of uses of the burs, no significant difference was found between the mean temperature values of the cases performed at the 1st use (Group 1 and Group 2) and 30th use (Group 3 and Group 4) (Independent T-test p=0.668)

The temperature changes in the sensors used in an example study group (Group 1) at depths of 5 and 10 mm are shown in Graph 1. In all groups, it was observed that the temperature values measured in both sensors were close to each other. It was also observed that there was no significant difference between the temperature values measured in the sensors

**Table 4:** Difference between the temperature values measured at Sensors 1 and 2 (S_1_, S)_2_^+^ Bonferroni analysis

Working Groups	Difference between measured values at the sensor (+ p)
Group 1	0.069
Group 2	0.611
Group 3	0.713
Group 4	0.708

### Milling cutter brands and temperature differences

Temperature differences were obtained by subtracting the initial temperatures from the temperatures measured in the working groups. The temperature differences and significance levels of different brands of milling cutters are given in Tables 5 and 6.

**Table 5:** Table showing milling cutter brands and temperature differences

	Ankylos		Astra Tech		Nobel Biocare		Bredent		Straumann		+ p
Group No	S_1_	S_2_	S_1_	S_2_	S_1_	S_2_	S_1_	S_2_	S_1_	S_2_	
1	5,11	6,10	3,73	3,58	3,98	4,17	5,08	6,97	3,12	4,75	0.137
2	0,42	3,08	0,52	2,60	5,45	5,03	0,02	0,57	3,77	0,72	0.191
3	6,05	7,33	2,28	5,55	7,18	4,35	4,08	5,90	1,63	2,62	0.176
4	3,28	4,92	4,08	6,27	3,23	2,00	5,72	3,03	3,20	5,55	0.496

^+^Kruskal Wallis Test

**Table 6:** Relationship between study groups and mean temperature differences

Group No	Number of Milling Cutter Uses	Mode of Operation	Revolutions (rpm)	Average Temperature Difference	+ p
1	First use	Without irrigation	150	27,7	,028*
2	First use	40 mL/min irrigation	1200	22,20	
3	30. Usage	Without irrigation	150	24,90	
4	30. Usage	40 mL/min irrigation	1200	21,20	

*Kruskal–Wallis test

The mean temperature changes in the study groups were found to be higher (p < 0.05). This indicates that the temperature values increased more in the non-irrigated study

## Discussion

In traumatic dental implant surgeries, connective tissue formation is seen around the implant during the healing process and this may cause failures in implant treatments. It is inevitable that the heat generated during implant drilling affects the living bone tissue in the implant placement area [8, 12]. It is thought that this heating occurs due to the friction between the bone tissue and the drill during drilling and the healing of the bone tissue is adversely affected at temperatures above 47 °C [13, 14]. The purpose of irrigation during drilling is to reduce the temperature increases that may occur in the bone [15]. When the temperature change graphs in the groups irrigated with 40 ml/min isotonic solution in our study were examined, it was observed that the temperature values on the bone surface were in the direction of decrease. On the other hand, it was observed that there would not be a significant temperature increase in the bone if irrigation was not applied at low cycles.Studies have shown that implant losses occur in the first year after implant placement at a high rate. It shows the importance of the surgical procedure to achieve success in implant applications [11]. Markovic et al. [16] placed a total of 288 *self-tapping* and *non-self-tapping* implants of Bredent and Straumann brands with torque forces of 30, 35 and 40 N in an in vitro study in porcine ribs. They measured the temperature changes observed at 1, 5 and 10 mm depths during placement. As a result of the study, they concluded that thermal effects would be observed less at low insertion torques in *self-tapping* implants. Trisi et al. [17] placed implants in the iliac crest of sheep at different temperatures in their in vivo study. They prepared a total of 15 implant sites in the study. During the opening of the implant socket, they kept the temperature of 5 of these sites at 50 °C for one minute and 5 of these sites at 60 °C for one minute. The remaining 5 implant sites were prepared without any temperature increase. No implant loss was observed in the study. However, they concluded that in the regions prepared at 60 °C for one minute, implant crestal bone loss increased in the later period after osteointegration and bone implant contact was less in this group. Sumer et al. [18] placed a total of 64 implants in bovine femur in their in vitro study. They divided 4.1 and 4.8 mm diameter implants into different groups and placed them manually at speeds of 30, 50 and 100 rpm. As a result of the study, they found that the highest temperature change (9.81 ± 2.29 °C) occurred in implants with a diameter of 4.1 mm and a speed of 100 rpm. As a result of the study, they argued that implant placement performed manually or at speeds of 30 and 50 rpm was safer than placement performed at 100 rpm. Allsobrook et al. [19] in an in vitro study on bovine head, Allsobrook et al. [19] examined the trauma caused by tungsten carbide and steel drills on the bone depending on the number of times they were used by SEM method. In all of their applications, they ensured that the temperature did not exceed 27.7 °C. After the study, they argued that the burs did not reach damaging temperature values even after 50 times of use. The data obtained from this study also showed that there was no significant change in the temperature increases in the bone after the first and 30th use of the burs. Chacon et al. [20] argued that the geometric structure of the burs and the wear caused by the use of the burs were effective in the temperature changes that occurred during the resurfacing process. In their study on cortical bone in bovine femur, they used a serum irrigated system with a speed of 2500 rpm and a constant force of 2.4 kg. Accordingly, they suggested that after 25 uses, the temperature value in the bone in *triple twist drills without a relief angle* can rise above 47 °C and healing may be impaired. This suggests that critical temperature increases may affect implant success. Matsuoka et al. [21] placed *self-drill* mini implants at speeds of 50, 100, 150, and 250 rpm into bone containing cortical layers of different thickness and observed the temperature change. They reported that the temperature increase was higher in the insertions made in the region where the cortical bone was thicker. At 250 rpm, they reported that temperature increases of more than 10 °C occurred, therefore, the instrument speed should be kept below 150 rpm in self-drilling miniscrew placements. In our study, the speed was set as 150 rpm in the groups without irrigation and it was observed that no significant temperature increase occurred in the bone when working at this speed.

Gaspar et al. [22] examined the temperature changes during the resurrection of a total of 36 implants placed in the rabbit tibia and the histologic changes observed in the early period. Accordingly, they reported that irrigation-free operation at 50 rpm and irrigated operation at 800 rpm gave approximate results in terms of temperature change. During osteotomy for implant applications, bone heating occurs due to the friction of the drill against the bone. To prevent this heating in the bone, the implant socket should be prepared by cooling with saline irrigation. Even if cooling is performed during bone preparation, some necrosis occurs around the implant socket. The size of the necrotic area depends on factors such as both heat and blood supply of the implanted area [23]. When necrosis occurs, the response of the bone to the necrotic area can be in 3 different ways:

1. Fibrous tissue formation: A certain amount of fibrous tissue forms in the bone, especially in cases of high trauma. The formation of fibrous tissue around the implant is easier than the formation of bone tissue.
2. Sequestration formation: If the blood supply to the tissue is insufficient and the surgery is traumatic, the bone necrosizes and does not heal.
3. New bone formation: Cortical bone formation around the implant is achieved with atraumatic surgery and adequate revascularization [24].

After implantation, remodeling around the implant is desired. Bone repair of necrotic implant cortex depends on the presence of sufficient number of cells in the area, adequate nutrition of these cells and sufficient stimulus for bone repair [25]. Many researchers have reported different opinions regarding the drilling speed during implant osteotomy. Albreksston et al. Albreksston et al. reported that the maximum drill speed should be 2000 revolutions during bone cavity preparation, whereas Babush et al. They reported 1500–1600 revolutions per minute in internally cooled milling systems, a maximum of 500 revolutions per minute in externally cooled systems and no more than 20 revolutions per minute during implant placement [7]. During implant osteotomy, if the bone tissue is exposed to a temperature higher than 43°C in one minute, a temperature that can cause denaturation of bone cells is reached. Since the high temperature causes alkaline phosphatase destruction in the bone and prevents calcium synthesis, new bone formation around the implant does not occur. Thus necrotic tissue forms around the implant. This situation prevents the formation of osteointegration and causes fibroosteosis integration [26].

Sandalli, on the other hand, stated that the quality of bone in different anatomical regions is different and a standard milling application cannot be sufficient, and the lowest speed that can cut the bone is the most appropriate speed [27]. It has been reported that when resistance is encountered while preparing the implant cavity, the pressure on the milling increases the heat generated in the bone and causes an increase in the amount of necrotic area. Other methods to reduce the high temperature that may occur during osteotomy include using a drill suitable for the implant system, using the drills sequentially, working under abundant irrigation, using a torque-adjustable physiodispenser and the sharpness of the drills used [26].As in bone, it is important that soft tissue surgery is atraumatic. The incision should be sharp and properly limited, and the mucoperiosteal flap should be lifted precisely. Maximum effort should be made not to damage the periosteum [28].

In the literature, bovine ribs were used in a significant number of in vitro studies in which temperature changes in bone were measured. In this study, bovine rib was used [29, 30]. In the studies, the similarity of the density of bovine ribs with the implanted bones was reported. In this study, it was macroscopically observed that the cortical/cancellous bone ratios in the bovine rib were similar to the mandible. On the other hand, in our study, it was seen that bovine ribs are easily obtainable at low costs and can also be made ready to work quickly.

There are also studies in the literature in which a device that applies constant force during drilling is used. Karaca et al. [31] prepared a device similar to the device in our study in their study in which they measured the effect of the drill diameter, speed of the drill and applied forces on temperature changes. As a result of their study, they reported that the temperature increased the most at the depth value between 4.5 mm and 6.5 mm and that the temperature increased more in titanium coated drills. In their study, they performed the drills in dry and extracellular tissue fluid-containing environments and reported that the environment in which the drills were made did not significantly affect the temperature changes. Oliveira et al. [32]. used bovine ribs in an in-vitro study in which they prepared an implant socket. They reported that there was no difference in temperature change between the first and 30th use of implant drills. In this study, they also observed a greater temperature increase in steel drills. In our study, the results obtained for the number of uses of the drills were similar to the results of this study. In cases where the amount of cortical bone is high, it is expected that the temperature increase during drilling will also be high. In their study, Abboud et al. [33] reported that the temperature increase is higher in cases with increased cortical bone density; on the other hand, the temperature will increase more with the prolongation of the drilling time. Based on this study, it should be kept in mind that the temperature increase may also increase in bones with high cortical thickness (Type 1 and Type 2).

## Conclusion

No significant difference was observed between the temperature changes in the bone during the operation of the drills manufactured by different companies and used in the preparation of the implant socket. This indicates that there is no significant difference in the implant drills used. In both the high-speed irrigated and the low-speed non-irrigated systems, the temperature values in the bone during drilling generally do not exceed the critical threshold of 47 °C. This demonstrates that both systems can be used safely by clinicians regardless of the manufacturer. However, it should be kept in mind that the temperature changes in non-irrigated systems are higher than in irrigated systems.

In the future, drilling procedures under different conditions can be performed to examine histologically the conditions that develop due to temperature increases in the bone.

## Data Availability

No datasets were generated or analysed during the current study.

## References

[1] Peker Tekdal G, Bostanci N, Belibasakis GN, Gurkan A. The effect of piezoelectric surgery implant osteotomy on radiological and molecular parameters of peri-implant crestal bone loss: a randomized, controlled, split-mouth trial. Clin Oral Implants Res. 2015. 10.1111/clr.12620 26077862

[2] dos Santos PL, Queiroz TP, Margonar R, de Souza Carvalho AC, Betoni W Jr, Rezende RR, et al. Evaluation of bone heating, drill deformation, and drill roughness after implant osteotomy: guided surgery and classic drilling procedure. Int J Oral Maxillofac Implants. 2014;29(1):51–8. 24451853 10.11607/jomi.2919

[3] Fugazzotto PA. Success and failure rates of osseointegrated implants in function in regenerated bone for 72 to 133 months. Int J Oral Maxillofac Implants. 2005;20(1):77–83. 15747677

[4] Anitua E, Begona L, Orive G. Controlled ridge expansion using a two-stage split-crest technique with ultrasonic bone surgery. Implant Dent. 2012;21(3):163–70. 22534365 10.1097/ID.0b013e318249f50b

[5] Thomas GE, Bone S, Drago G. Determination of protein denaturation and glass transition temperatures using high-frequency time domain reflectometry. J Phys Chem B. 2008;112(49):15903–6. 19368035 10.1021/jp806775w

[6] Trebacz H, Wojtowicz K. Thermal stabilization of collagen molecules in bone tissue. Int J Biol Macromol. 2005;37(5):257–62. 16414113 10.1016/j.ijbiomac.2005.04.007

[7] Brisman DL. The effect of speed, pressure, and time on bone temperature during the drilling of implant sites. Int J Oral Maxillofac Implants. 1996;11(1):35–7. 8820120

[8] Ast MP, Cabrera BJ, DiMaio FR, Lementowski P. Cold saline lavage for removal of incarcerated porous ingrowth stems. Orthopedics. 2011;34(12):e936–8. 22146213 10.3928/01477447-20111021-30

[9] Markovic A, Misic T, Mancic D, Jovanovic I, Scepanovic M, Jezdic Z. Real-time thermographic analysis of low-density bone during implant placement: a randomized parallel-group clinical study comparing lateral condensation with bone drilling surgical technique. Clin Oral Implant Res. 2014;25(8):910–8. 10.1111/clr.12191 23710900

[10] Lucchiari N, Frigo AC, Stellini E, Coppe M, Berengo M, Bacci C. In vitro assessment with the infrared thermometer of temperature differences generated during implant site preparation: the traditional technique versus the single-drill technique. Clin Implant Dent Relat Res. 2014. 10.1111/cid.12246. 25040939 10.1111/cid.12246

[11] Eriksson AR, Albrektsson T. Temperature threshold levels for heat-induced bone tissue injury: a vital-microscopic study in the rabbit. J Prosthet Dent. 1983;50(1):101–7. 6576145 10.1016/0022-3913(83)90174-9

[12] Albrektsson T, Eriksson A. Thermally induced bone necrosis in rabbits: relation to implant failure in humans. Clin Orthop Relat Res. 1985;195:311–2. 3978966

[13] Eriksson AR, Albrektsson T, Albrektsson B. Heat caused by drilling cortical bone. Temperature measured in vivo in patients and animals. Acta Orthopaed Scand. 1984;55(6):629–31. 10.3109/17453678408992410 6524329

[14] Eriksson A, Albrektsson T, Grane B, McQueen D. Thermal injury to bone. A vital-microscopic description of heat effects. Int J Oral Surg. 1982;11(2):115–21. 6809671 10.1016/s0300-9785(82)80020-3

[15] Li CH, Chou CT. Bone sparing implant removal without trephine via internal separation of the titanium body with a carbide bur. Int J Oral Maxillofac Surg. 2014;43(2):248–50. 24176547 10.1016/j.ijom.2013.09.010

[16] Markovic A, Misic T, Milicic B, Calvo-Guirado JL, Aleksic Z, Ethinic A. Heat generation during implant placement in low-density bone: effect of surgical technique, insertion torque and implant macro design. Clin Oral Implant Res. 2013;24(7):798–805. 10.1111/j.1600-0501.2012.02460.x 22469169

[17] Trisi P, Berardini M, Falco A, Vulpiani MP. Effect of temperature on the dental implant osseointegration development in low-density bone: an in vivo histological evaluation. Implant Dent. 2015;24(1):96–100. 25621555 10.1097/ID.0000000000000204

[18] Sumer M, Keskiner I, Mercan U, Misir F, Cankaya S. Assessment of heat generation during implant insertion. J Prosthet Dent. 2014;112(3):522–5. 24656407 10.1016/j.prosdent.2013.12.011

[19] Allsobrook OF, Leichter J, Holborrow D, Swain M. Descriptive study of the longevity of dental implant surgery drills. Clin Implant Dent Relat Res. 2011;13(3):244–54. 22106473 10.1111/j.1708-8208.2009.00205.x

[20] Chacon GE, Bower DL, Larsen PE, McGlumphy EA, Beck FM. Heat production by 3 implant drill systems after repeated drilling and sterilization. J Oral Maxillofacial Surg. 2006;64(2):265–9. 10.1016/j.joms.2005.10.011 16413899

[21] Matsuoka M, Motoyoshi M, Sakaguchi M, Shinohara A, Shigeede T, Saito Y, et al. Friction heat during self-drilling of an orthodontic miniscrew. Int J Oral Maxillofac Surg. 2011;40(2):191–4. 21094024 10.1016/j.ijom.2010.10.007

[22] Gaspar J, Borrecho G, Oliveira P, Salvado F, Martins dos Santos J. Osteotomy at low-speed drilling without irrigation versus high-speed drilling with irrigation: an experimental study. Acta Medica Portuguesa. 2013;26(3):231–6. 23815837

[23] Von Steenberghe D, Branemark PI, Quirynen M, De Mars G, Noert I. The rehabilitation of oral defects by osseointegrated implants. J Clin Periodontol. 1991;18:488–93. 1890233 10.1111/j.1600-051x.1991.tb02321.x

[24] Albrektsson T, Branemark PI, Zarb G. Tissue integrated prostheses. Chicago: Quintessence Publishing; 1995. p. 199–209.

[25] Kim SJ, Yoo J, Kim YS, Shin SW. Temperature change in pig rib bone during implant site preparation by low-speed drilling. J Appl Oral Sci. 2010;18(5):522–7. 21085811 10.1590/S1678-77572010000500016 PMC4246386

[26] Von Recum AF, Schreuders PD, Powers DL. Basic healing phenomena around permanent percutaneous implants. In: Van Steenberghe (ed). Proceedings of the International Congress of Tissue Integration in Oral and Maxillofacial Reconstruction. Amsterdam: Excerpta Medica, 1986:159–169

[27] Sandalli P: Success and failure in oral implantology. ICOI XV World Congress, San Francisco, California,2005.

[28] Al-Juboori MJ, Bin Abdulrahaman S, Dawood HF. Principles of flap design in dental implantology. Dent Implantol Update. 2012;23(6):41–4. 22693764

[29] In Vitro Assessment with the Infrared Thermometer of Temperature Differences Generated During Implant Site Preparation: The Traditional Technique Versus the Single-Drill Technique. 10.1111/cid.12246 25040939

[30] Scarano A, Carinci F, Quaranta A, Di Iorio D, Assenza B, Piattelli A. Effects of bur wear during implant site preparation: an in vitro study. Int J Immunopathol Pharmacol. 2007;20(1 Suppl 1):23–6. 17897497 10.1177/039463200702001s06

[31] Karaca F, Aksakal B. Effects of various drilling parameters on bone during implantology: an in vitro experimental study. Acta Bioeng Biomech. 2013;15(4):25–32. 24479623

[32] Olivera N, Algarra FA, Bueno JM, Padro EF, Alfaro FH. Thermal changes and drill wear in bovine" bone during implant site preparation A comparative in vitro study: twisted stainless steel and ceramic drill 10.1111/j.1600-0501.2011.02248.x 21806686

[33] Abboud M, Delgado-Ruiz RA, Kucine A, Rugova S, Balanta J, Calvo-Guirado JL. Multistepped drill design for single-stage implant site preparation: experimental study in type 2 bone. Clin Implant Dent Relat Res. 2015. 10.1111/cid.12273. 25263993 10.1111/cid.12273

[34] Şarkbay ÖF. *Farklı Marka İmplant Frezleri ile Yapılan Osteotomilerde Kemikte Meydana Gelen Sıcaklık Değişimlerinin In-Vitro Olarak İncelenmesi.* Master’s thesis, Bezmialem Vakıf Üniversitesi, 2015.

